# Development of a High-Throughput Respiratory Syncytial Virus Fluorescent Focus-Based Microneutralization Assay

**DOI:** 10.1128/CVI.00225-17

**Published:** 2017-12-05

**Authors:** Cindy Shambaugh, Sarieh Azshirvani, Li Yu, Jared Pache, Stacie L. Lambert, Fengrong Zuo, Mark T. Esser

**Affiliations:** a Translational Sciences, MedImmune, Mountain View, California, USA; b Statistical Sciences, MedImmune, Gaithersburg, Maryland, USA; c Molecular Devices, Sunnyvale, California, USA; d Translational Medicine, MedImmune, Gaithersburg, Maryland, USA; Parasitology Services

**Keywords:** RSV A green fluorescent protein, fluorescent focus-based microneutralization assay, high-content image analysis, neutralizing antibodies, respiratory syncytial virus, vaccines, virus neutralization

## Abstract

Neutralizing antibodies specific for respiratory syncytial virus (RSV) represent a major protective mechanism against RSV infection, as demonstrated by the efficacy of the immune-prophylactic monoclonal antibody palivizumab in preventing RSV-associated lower respiratory tract infections in premature infants. Accordingly, the RSV neutralization assay has become a key functional method to assess the neutralizing activity of serum antibodies in preclinical animal models, epidemiology studies, and clinical trials. In this study, we qualified a 24-h, fluorescent focus-based microneutralization (RSVA FFA-MN) method that requires no medium exchange or pre- or postinfection processing to detect green fluorescent protein-expressing RSV strain A2 (RSVA-GFP)-infected cells, using a high-content imaging system for automated image acquisition and focus enumeration. The RSVA FFA-MN method was shown to be sensitive, with a limit of detection (LOD) and limit of quantitation (LOQ) of 1:10, or 3.32 log_2_; linear over a range of 4.27 to 9.65 log_2_ 50% inhibitory concentration (IC_50_); and precise, with intra- and interassay coefficients of variation of <21%. This precision allowed the choice of a statistically justified 3-fold-rise seroresponse cutoff criterion. The repeatability and robustness of this method were demonstrated by including a pooled human serum sample in every assay as a positive control (PC). Over 3 years of testing between two laboratories, this PC generated data falling within 2.5 standard deviations of the mean 98.7% of the time (*n* = 1,720). This high-throughput and reliable RSV microneutralization assay has proven useful for testing sera from preclinical vaccine candidate evaluation studies, epidemiology studies, and both pediatric and adult vaccine clinical trials.

## INTRODUCTION

Respiratory syncytial virus (RSV) is the most common cause of lower respiratory tract infections (LRTIs) in children under the age of 2 years; however, it can cause severe illness in infants with underdeveloped lungs, the elderly, and those with chronic heart or lung diseases (1). Currently, there are no licensed vaccines available to prevent RSV infection, though immunoprophylaxis using an RSV neutralizing monoclonal antibody, palivizumab, has been available for high-risk infants since 1998 (2). The development of a vaccine requires the ability to measure immunogenicity in preclinical animal models, epidemiology studies, and vaccine clinical trials. Vaccine immunogenicity can be assessed by measuring biomarkers, such as virus-neutralizing serum antibodies, RSV F- or G-specific serum antibodies, or cellular immune responses, including RSV antigen-specific T and B cells. The nature of the vaccine and the clinical target population often decide the serological methods used to measure protective immunity.

While protection from RSV infection is not completely understood, it is thought that neutralizing antibodies present at or above a protective threshold would prevent infection and serious disease (3, 4). Passive immunization with high-titer intravenous immunoglobulin (IVIG) or RSV-neutralizing monoclonal antibodies (MAbs) reduces serious disease caused by RSV. Thus, the neutralization assay is considered one of the key functional methods of measuring protective immunity elicited by vaccine candidates. Historically, the plaque reduction neutralization test (PRNT) has been the gold standard for measurement of RSV-neutralizing activity, although several more efficient RSV microneutralization (MN) assays have been developed (5–9). While some of these methods have short incubation times, most require one of the following: a virus inoculum medium exchange, postinfection processing, or multiple days of incubation to perform an assay.

In this report, we discuss the development and qualification of a simple, high-throughput, and robust automated RSV A fluorescent focus-based microneutralization (RSVA FFA-MN) assay. Using this assay, more than 100 samples can be tested per day per analyst, without any pre- or postincubation processing. The qualification of the assay and control trending data demonstrated that this RSVA FFA-MN assay has the sensitivity, linearity, precision, reproducibility, and robustness that are required to detect RSV-specific neutralizing antibodies elicited following natural infection or vaccination.

## RESULTS

### Optimization of RSVA-GFP input concentration and infection incubation time.

**FIG 1:**
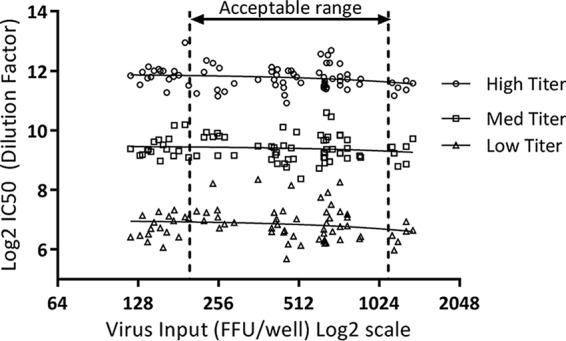
Effect of RSVA-GFP concentration on determination of neutralizing antibody titers. Virus concentrations ranging from 100 to 1,400 (6.64 to 10.45 log_2_) FFU/well were used to test serum samples with high, medium, and low neutralizing antibody titers.

To determine the optimal concentration of an RSV strain A2 recombinant virus expressing green fluorescent protein (RSVA-GFP) to add to the assay, the RSVA-GFP stock virus was diluted to cover a range of concentrations that would be quantifiable in a 96-well plate by use of an imaging system. Virus input concentrations ranging from 100 to 1,400 fluorescent focus-forming units (FFU)/well were used in the neutralization assay to test human serum that had been pooled into high-, medium-, and low-neutralizing-titer samples. Neutralization titers were plotted against log_2_ virus input concentrations to calculate the linear regression analysis for each sample (Fig. 1). These data show that there was no statistically significant trend in antibody titers across a range of virus input concentrations for low-, medium-, and high-titer serum samples (*P* > 0.05). Acceptable assay parameters were set to tolerate an input virus range of 200 to 1,100 FFU/well (multiplicity of infection [MOI] range, 0.01 to 0.06). While the tolerance was set for wider acceptance criteria, the targeted virus input was set at 600 ± 100 FFU/well.

**FIG 2:**
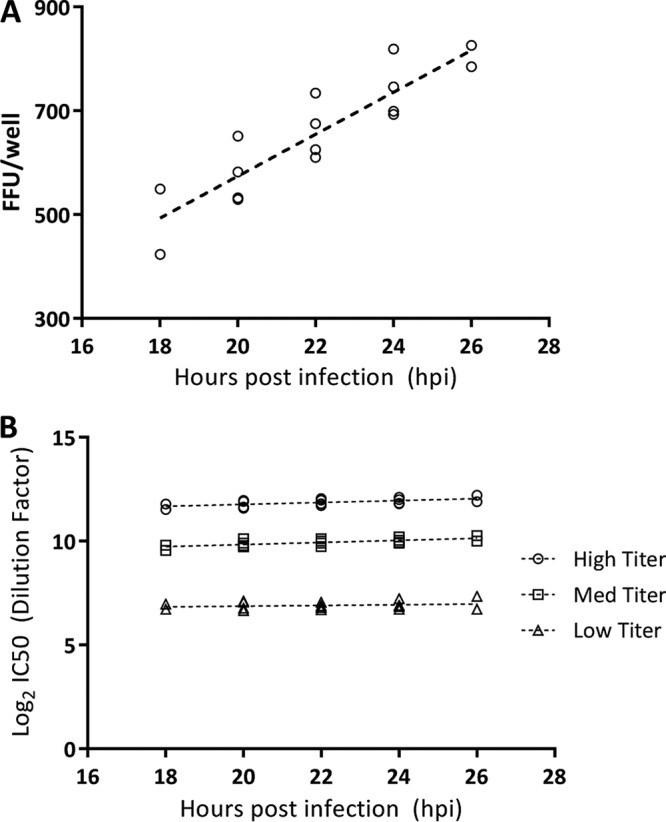
Effects of microneutralization incubation time on virus infection and RSV MN antibody titers. (A) Effect of RSV-GFP incubation time on the number of fluorescent foci. The number of foci counted per well increased with increased incubation times of the FFA-MN assay. (B) Effect of serum and RSV-GFP incubation time on RSV MN antibody titers. The data show comparisons of high-, medium-, and low-titer serum samples over a range of incubation times (18 to 26 h).

We next evaluated the impacts of different incubation times of RSVA-GFP and serum on Vero cells in 96-well plates. In earlier studies, RSVA-GFP growth in Vero cells was measured by counting foci at various time points over a 48-h period by use of an IsoCyte laser imager. The virus infection level for a virus input concentration of ∼600 foci/well (MOI = 0.03) increased linearly from 15 to 25 h postinfection (hpi), with no new foci detected after 27 hpi (see Fig. S1 in the supplemental material). Focus numbers appeared to drop off after 28 hpi, but plate well images revealed that a majority of foci had increased in size and begun to fuse with neighboring foci, demonstrating viral spread from the initial infected cell rather than cell loss. Based on these data, we further investigated the window from 18 to 26 hpi to determine the optimal time point for focus quantitation of RSVA-GFP with serum. The number of foci detected increased in a linear fashion with longer incubation times (Fig. 2A). Three serum samples, with high, medium, and low neutralizing titers, were used to analyze single-round infections for up to 26 h postinfection. Linear regression analysis was used to evaluate the trend of neutralizing titers (*n* = 4) for each sample over time, with no significant change (*P* > 0.05) found for the range of 20 to 26 h (Fig. 2B). Based on these data, the assay incubation parameter was set to 22 to 24 h. At 22 to 24 h, the individual control viral foci were easily distinguished either by GFP signal or by immunostaining following acetone fixation (10), with similar enumerations of foci by the different methods (259 versus 250 foci, respectively) (Fig. S2).

### Correlation of RSVA FFA-MN and PRNT results.

**FIG 3:**
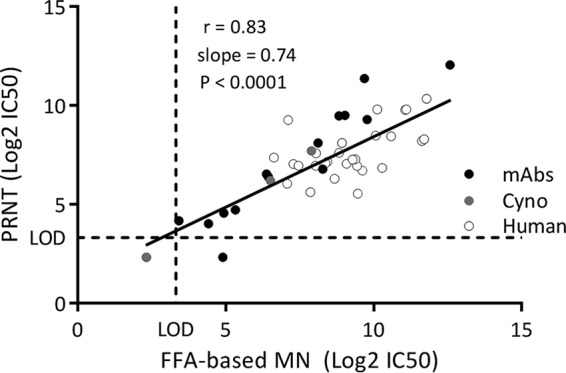
Comparison of RSVA-GFP PRNT and RSVA-GFP FFA-MN assay by use of a panel of 15 MAbs and 31 serum samples. Samples included individual and pooled human sera and cynomolgus macaque (Cyno) sera.

A panel of 15 MAbs and 31 serum samples derived from individual and pooled human sera as well as RSV-immunized cynomolgus macaque sera was used to compare the RSVA FFA-MN assay to a PRNT. Neutralizing titers of the panel ranged from <3.32 to 12.58 log_2_ 50% inhibitory concentration (IC_50_). A value of 2.32 log_2_ (half the limit of detection [LOD] of the assay) was imputed for samples with titers of <3.32 log_2_. A plot of the RSV FFA-MN versus PRNT results shows a correlation coefficient (*r*) of 0.83, with a slope of 0.74 (*P* < 0.0001) (Fig. 3).

### Automation.

The RSVA FFA-MN assay was streamlined by implementing a Bravo SRT automated liquid handler (Agilent, Santa Clara, CA) for the following two steps within the assay protocol: (i) sample serial dilution and (ii) transfer of virus-serum mixtures to washed Vero cell plates. The Bravo SRT handler was installed in a biological safety cabinet (BSC) to protect samples and provide environmental protection. This automation allowed for a higher sample throughput and reduced variability due to manual operator pipetting. Additionally, plate image acquisition was semiautomated using the MetaXpress software associated with an ImageXpress Micro XLS (IXM) high-content imaging system. MetaXpress software acquires plate image data in <2 min and subsequently analyzes the well images by using settings developed in the custom module editor, which uses filters and masks to define the foci, detected as objects by use of a “count nuclei” feature. The IXM system continues to image new plates while data are analyzed and concurrently logged into a validated data analysis spreadsheet through the use of an autorun program in MetaXpress. Alternatively, a robotic arm may be implemented to deliver plates into the IXM system for fully automated plate reading. The data analysis template was developed and validated in-house to contain embedded links and formulas that calculate the log_2_ IC_50_ titer for 2 replicate samples. The automated transfer of focus counts into the data analysis template eliminated the need to manually transfer data from the instrument to an Excel spreadsheet, thereby simplifying the process and avoiding potential errors of a copy-and-paste method.

### Assay qualification.

The precision of the RSVA FFA-MN assay was determined by testing of pooled human serum in 2 replicates in 2 assays by 2 analysts over 4 days, for a total of 32 data points. Intra-assay precision was 20.9%, interassay precision was 11.7% and total assay precision was 24.1% (Table 1). A subsequent precision analysis using four individual human serum samples covering a range of titers, from 4.20 to 13.28 log_2_, was performed by 2 analysts over 4 days. Each sample gave an overall precision of ≤24.1%, consistent with the qualification data (Table 1). Additionally, we evaluated the virus control variability by calculating the mean virus titer for 32 plates run over 8 different days by 2 analysts during assay qualification at the contract research organization (CRO). The mean virus titer was 568 FFU/well, with a coefficient of variation (CV) of 13.2% (data not shown).

**TABLE 1:** Precision data for qualification of the RSVA FFA-MN assay^*a*^

Sample	IC_50_ GMT	Mean log_2_ IC_50_ titer	No. of replicates	%CV		
				Intermediate (interassay)	Repeatability (intra-assay)	Total
Pooled human serum	869	9.76	32	11.7	20.9	24.1
Individual human sera						
1	24	4.58	28	5.2	10.7	11.9
2	268	8.07	28	7.5	13.6	15.5
3	879	9.78	28	6.0	20.8	21.7
4	6,840	12.74	21	7.8	22.7	24.1

a The precision of the assay was determined using pooled human serum, and the testing included 2 analysts, 2 assays, and 2 replicates over 4 days of testing, for a total of 32 data points. Total assay variability was 24.1% (%CV). Four individual human serum samples with titers ranging from 24 to 6,840 IC_50_ were all within the acceptable range for total assay variability.

**FIG 4:**
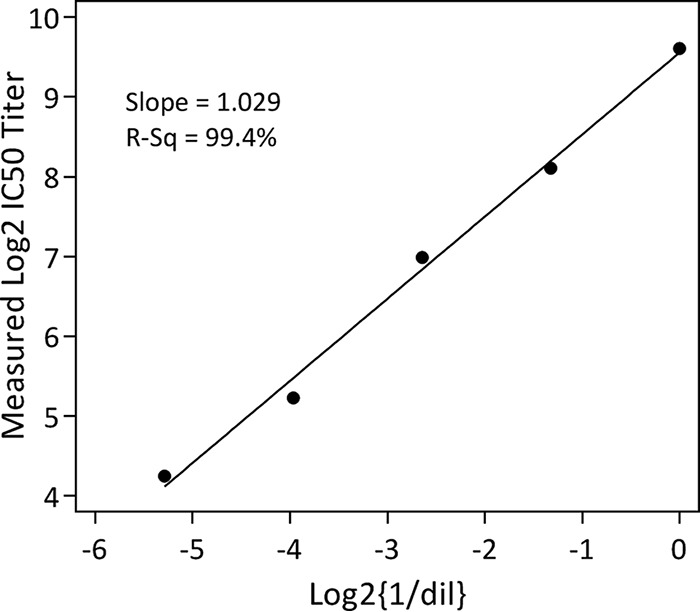
RSVA FFA-MN assay linearity. A linear model is shown for 5 samples diluted individually in 2.5-fold increments. These data were generated by 2 analysts, 2 assays, and 4 days of testing to obtain 80 data points, giving an *R*^2^ value of 99.4% and a slope of 1.029.

A linearity study was conducted in which pooled human serum at 9.65 log_2_ IC_50_ was diluted in 2.5-fold increments to generate 5 samples. These 5 samples were tested for precision in 2 assays by 2 analysts over 4 days of testing, for a total of 80 data points, with total assay variability (%CV) ranging from 20.7 to 30.2% (Table 2). The fitted linear model resulted in a slope estimate of 1.0 ± 0.1, showing linearity of this assay in the range of 4.27 to 9.65 log_2_ IC_50_ (Fig. 4).

**TABLE 2:** Individual results from the linearity study

Sample	Mean log_2_ IC_50_ titer	IC_50_ GMT	Assay total %CV^*a*^
S1	9.65	801.1	20.7
S2	8.17	287.4	27.5
S3	6.64	100.1	30.2
S4	5.17	35.9	26.5
S5	4.27	19.2	21.8

a Overall total assay variability was <31%.

Given the precision and linearity of the assay, it was determined that there was a <1% probability that a 3-fold difference in antibody titer from one subject at two time points could be due to assay variability. Thus, a 3-fold rise was identified as the cutoff for a true seroresponse in subjects who may have been vaccinated or experienced an RSV infection.

**FIG 5:**
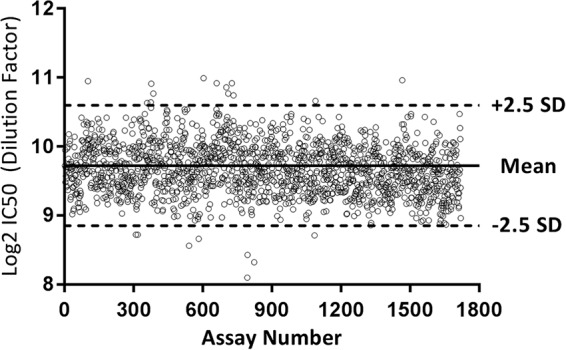
Positive-control (PC) trend. The chart displays IC_50_ neutralization data for each individual assay plate run for the RSVA FFA-MN assay. PC data shown were collected for more than 2.5 years by 7 analysts in 2 separate laboratories (*n* = 1,720) and demonstrate the assay robustness by the large number of data points (98.7%) that fell within 2.5 standard deviations of the mean.

A pooled human serum sample was used as a positive control (PC) and was included on each plate to monitor assay performance. PC data collected over approximately 3 years of testing by 7 analysts in 2 separate laboratories produced over 1,700 data points, with a %CV of 28.6%. Assay robustness was confirmed by the high percentage (98.7%) of PC data points that fell within 2.5 standard deviations of the mean (Fig. 5).

## DISCUSSION

The RSV neutralization assay is an important functional assay to detect serum antibodies that may serve as biomarkers of vaccine efficacy. There are currently multiple approaches for determining RSV neutralization titers that cover a wide range of detection methods (5–9), including those using a GFP-expressing virus. Many of these neutralization assays possess characteristics that are desirable for use in clinical trials, such as high sensitivity, reproducibility, increased throughput, and amenability to some automation (Table 3). What sets the RSVA FFA-MN method apart is a streamlined process that does not require any pre- or postinfection incubation processing or manual data manipulation. The 96-well plates go directly from incubator to image acquisition and subsequent data analysis by use of a validated Excel spreadsheet. Less plate handling minimizes assay variability and improves sample throughput. Another benefit of the RSVA FFA-MN method described here is that it can easily be adapted to be run without the use of a GFP-labeled virus to test serum neutralization of different clinical virus isolates by immunostaining the fixed cell monolayer postinfection (10).

**TABLE 3:** Comparison of various RSV neutralization assay methods

Neutralization assay	Cell type	Preincubation steps	Incubation time	Postincubation steps	Advantage(s)	Source or reference
Flow cytometry-based assay	HEp-2	None	18 h	Trypsin treatment of cells to detach, fixing of cells	Rapid, sensitive, reproducible	5
Luciferase reporter-based assay	A549	None	16 h	Cell washing, cell lysis (15 min), addition of substrate (automated)	Simple, high throughput, automatable	6
Fluorescence-based assay	Vero	Spin inoculation and 37°C incubation (2 h), medium exchange with methyl cellulose	2 days	None	High throughput	7
Quantitative PCR (qPCR)-based assay	Vero	None	24 h	Wash cells and lyse, transfer lysate, add PCR reagents, run PCR	Sensitive, high throughput	8
Colorimetric automated plaque counting	Vero	Spin inoculation and rocking at RT (1.5 h), medium exchange with methyl cellulose	3 days	Fixing of cells, immunostaining (2+ h)	Semiautomated	9
FFA-based assay	Vero	None	22 h	None	Rapid, sensitive, reproducible, high throughput, semiautomated	MedImmune

The RSVA FFA-MN method was found to be robust, precise, and linear, with a high-throughput capacity of >100 samples per day (108 samples tested on 24 96-well plates) on an IXM system. The assay can be used for sera from multiple species and has proven utility in testing sera from epidemiology studies, preclinical vaccine candidate evaluation studies, and both pediatric and adult vaccine studies. In epidemiology studies, it could differentiate pre- and postinfection serum samples from subjects confirmed to have had an RSV infection by PCR (11). In evaluating vaccines in rodents, it helped to select adjuvants that could boost protective neutralizing antibody titers (12). In cynomolgus macaque and human adult vaccine studies, it showed the utility of a Toll-like receptor 4 agonist (glucopyranosyl A) in a stable emulsion (GLA-SE adjuvant) in increasing the immunogenicity of RSV sF-based vaccines (10, 13). Finally, this method was compared in an RSV neutralization assay survey study with 11 other labs by testing a panel of 57 samples that covered a wide range of neutralization titers (14) and was shown to correlate well with other single-cycle infection assays as well as multicycle infection assays.

## MATERIALS AND METHODS

### Construction of recombinant RSV-A2 expressing GFP.

A plasmid DNA sequence expressing enhanced green fluorescent protein (EGFP) (Clontech, Mountain View, CA) was inserted into wild-type (wt) RSV strain A2 at a genomic location where no RSV genes or their promoters were disrupted and where it would be under the control of the RSV gene start and gene stop transcriptional signal. After the RSV-A2-GFP (RSVA-GFP) recombinant virus was isolated, PCR amplification and sequencing were performed to confirm the GFP gene location. Viral stocks of RSVA-GFP were expanded in HEp-2 cells, formulated in Opti-MEM plus 2 mM l-glutamine with 1× sucrose phosphate buffer (HyClone), aliquoted into single-use volumes, and stored at −80°C until use (Aragen Bioscience, Morgan Hill, CA). The virus was later titrated at MedImmune in a 24-h fluorescent focus assay (FFA) to determine a virus concentration of 3.95 × 10^6^ FFU/ml.

### Cells.

Vero African green monkey kidney cells (ATCC CCL-81; ATCC, Manassas, VA) were maintained in 1× Dulbecco's modified Eagle's medium (DMEM) (Life Technologies, Grand Island, NY) supplemented with 5% γ-irradiated fetal bovine serum (FBS) (HyClone, Logan, UT), 4 mM l-glutamine (Life Technologies, Grand Island, NY), 100 μg/ml penicillin-streptomycin mixture (Life Technologies), and 1× nonessential amino acids (NEAA) (Sigma, St. Louis, MO). Cells between passages 123 and 147 were seeded 2 days prior to use in 96-well Costar tissue culture plates at 2.0 × 10^4^ cells/well and 200 μl/well to achieve a 95 to 100% confluent monolayer or in 24-well tissue culture plates at 1.5 × 10^5^ cells/well and 1 ml/well to achieve an 80 to 90% confluent monolayer for plaque reduction assays.

### Serum samples and controls.

Pooled normal human serum (Innovative Research, Novi, MI) with a mean microneutralization titer of 9.72 log_2_ IC_50_ was used as a positive control (PC) on each sample plate to evaluate assay performance. Prior to use in the assay, the pooled serum was heat inactivated at 56°C for 45 min, aliquoted, and stored at −80°C for individual assay use. Monoclonal antibodies were purchased from EMD Millipore (Billerica, MA) or were acquired internally. Human sera with RSV-neutralizing activity from natural RSV infections were purchased from Bioreclamation IVT (Hicksville, NY). Cynomolgus macaque sera were obtained from preclinical studies of RSV vaccine candidates (10). All samples were heat inactivated at 56°C for 45 to 60 min to remove complement activity and then stored at −80°C prior to testing.

### RSVA-GFP FFA-based microneutralization assay.

Samples and control sera were thawed at room temperature and diluted 1:10 to 1:21,870 in an 8-point, 3-fold-serial-dilution series in virus growth medium (VGM), consisting of minimal essential medium with Earle's balanced salt solution (MEM/EBSS) (HyClone), 2 mM l-glutamine (Life Technologies), 1× penicillin-streptomycin mixture (Life Technologies), and 1× NEAA (Sigma). Serial dilutions were performed using a Bravo SRT automated liquid handler (Agilent Technologies, Santa Clara, CA). RSVA-GFP was thawed quickly in a 37°C water bath, gently mixed by inversion, and diluted in VGM to a target concentration that produced approximately 500 to 700 FFU per well. Equal volumes of RSVA-GFP and serum or VGM were incubated at room temperature (RT) for 1 h for neutralization. The virus-serum mixture was added to prewashed Vero cells in 96-well plates by use of a Bravo SRT liquid handler to deliver 100 μl/well to each of two duplicate plates. Plates were incubated at 37°C and 5% CO_2_ for 22 to 24 h prior to image acquisition on an ImageXpress Micro XLS (IXM) machine (Molecular Devices, Sunnyvale, CA), using a 4× Plan Apo objective and a fluorescein isothiocyanate (FITC) fluorescence filter set with excitation and emission wavelengths of 482 and 536 nm. Fluorescent foci were enumerated by use of MetaXpress software, using a custom analysis module. The data were successively logged by the MetaXpress software into an Excel data analysis spreadsheet that was developed with embedded functions to calculate the log_2_ IC_50_ MN titer (fold dilution) for each sample and positive control without operator input. The lower limit of detection and lower limit of quantitation are both a 1:10 dilution of serum samples due to serum matrix interference at lower dilutions. Samples that produced results of >21,870 IC_50_ were retested using a higher starting dilution.

### Plaque reduction neutralization test.

Serum and PC sera were diluted as described above and mixed 1:1 with RSVA-GFP diluted to a concentration that would produce approximately 50 to 75 PFU/well in 24-well plates. After 1 h of incubation at RT, 100 μl of serum-virus mixture was added to Vero cell monolayers that had been washed with warmed VGM. The mixture was incubated at 37°C and 5% CO_2_ for 1 h before the inoculum was removed and cells were overlaid with ∼500 μl 1% methyl cellulose in L-15–Eagle's minimal essential medium (EMEM) (SAFC) supplemented with 2% FBS, 1× NEAA, 2 mM l-glutamine, and 1× penicillin-streptomycin. The plates were incubated at 37°C and 5% CO_2_ for 3 to 5 days. Fluorescent plaques were counted using an IsoCyte laser-based imaging system (Molecular Devices, Sunnyvale, CA).

### Data analysis.

Both the RSVA FFA-MN assay and the PRNT used the following calculation to determine the IC_50_ geometric mean titer (GMT). The virus control without serum was used to normalize focus count data, which were then used to calculate the IC_50_ (fold dilution) value in the presence of serum by using a 2-point interpolation from the points surrounding a 50% reduction in virus. The final neutralization titers were calculated from the GMT of IC_50_ titers for duplicate samples on two different cell plates and reported as log_2_ IC_50_ GMT.

## Supplementary Material

Supplemental material
