# Supplementary material for: Development of a High-Throughput Respiratory Syncytial Virus Fluorescent Focus-Based Microneutralization Assay
Source: Clin Vaccine Immunol. 2017 Dec 5;24(12):e00225-17. doi: 10.1128/CVI.00225-17 (PMC5717189; doi:10.1128/CVI.00225-17)
Supplement: Supplemental material [file supp_24_12_e00225-17__index.html]

Supplemental material 

# Development of a High-Throughput Respiratory Syncytial Virus Fluorescent Focus-Based Microneutralization Assay

## Supplemental material

- Supplemental file 1 -

  Fig. S1. RSVA-GFP growth curve in Vero cells as measured by fluorescent foci in 96-well plates using an IsoCyte laser imager.

  PDF, 134K
- Supplemental file 2 -

  Fig. S2. Screen shots from ImageXpress Micro MetaXpress software showing fluorescent foci per region of interest.

  PDF, 8.9M
- Supplemental file 3 -

  Legends for Fig. S1 and S2.

  PDF, 89K
