# Supplementary figures and images for: Development of a High-Throughput Respiratory Syncytial Virus Fluorescent Focus-Based Microneutralization Assay
Source: Clin Vaccine Immunol. 2017 Dec 5;24(12):e00225-17. doi: 10.1128/CVI.00225-17 (PMC5717189; doi:10.1128/CVI.00225-17)

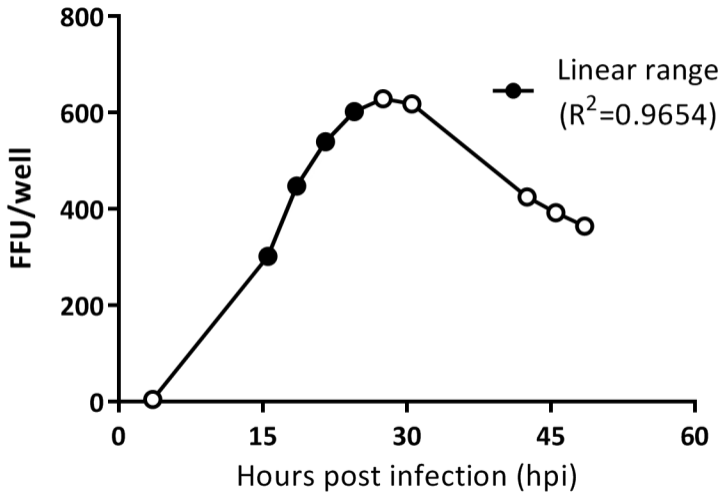

Supplement: Supplemental material [file CVI.00225-17_zcd012175545s1.pdf]

A

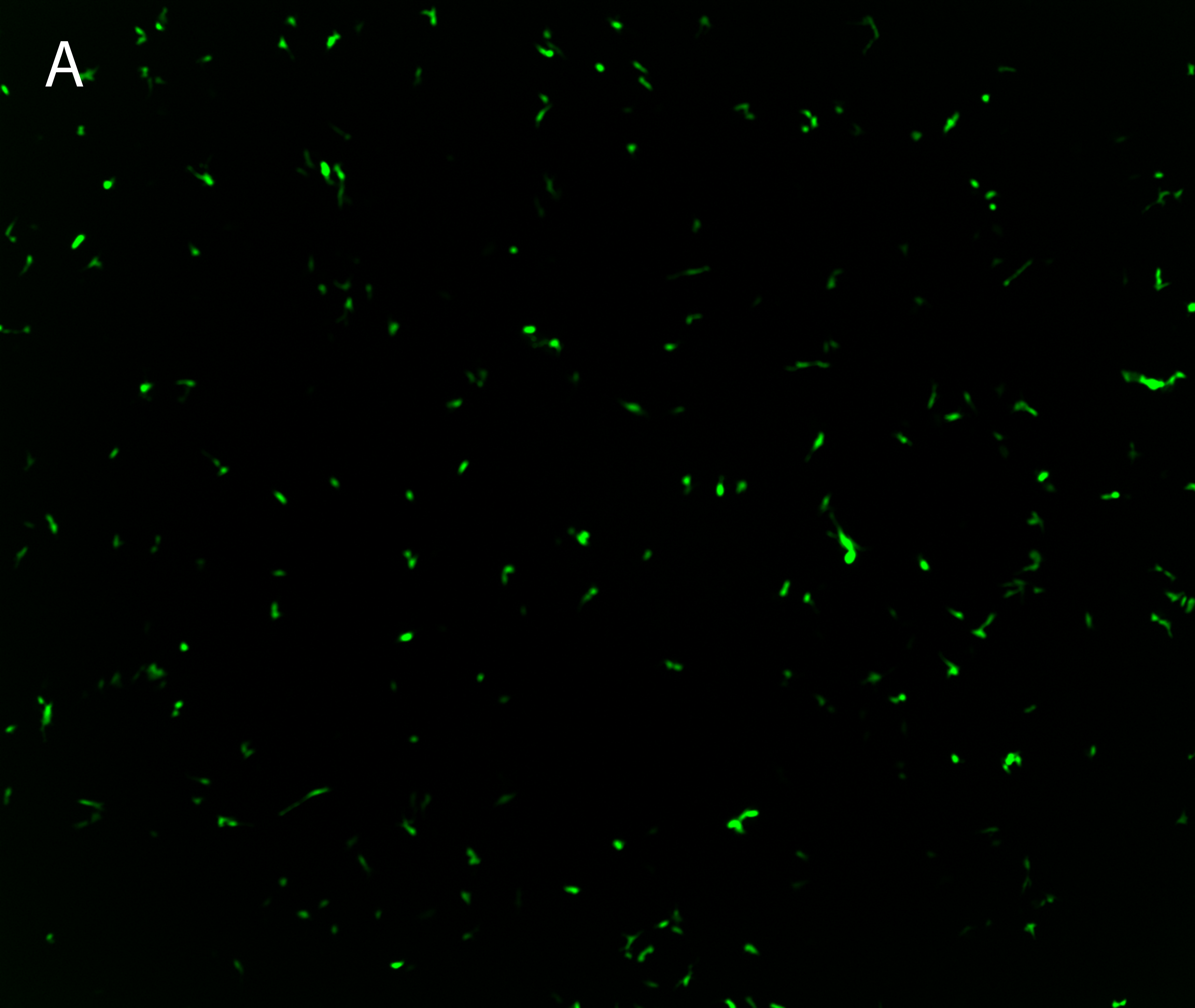

B

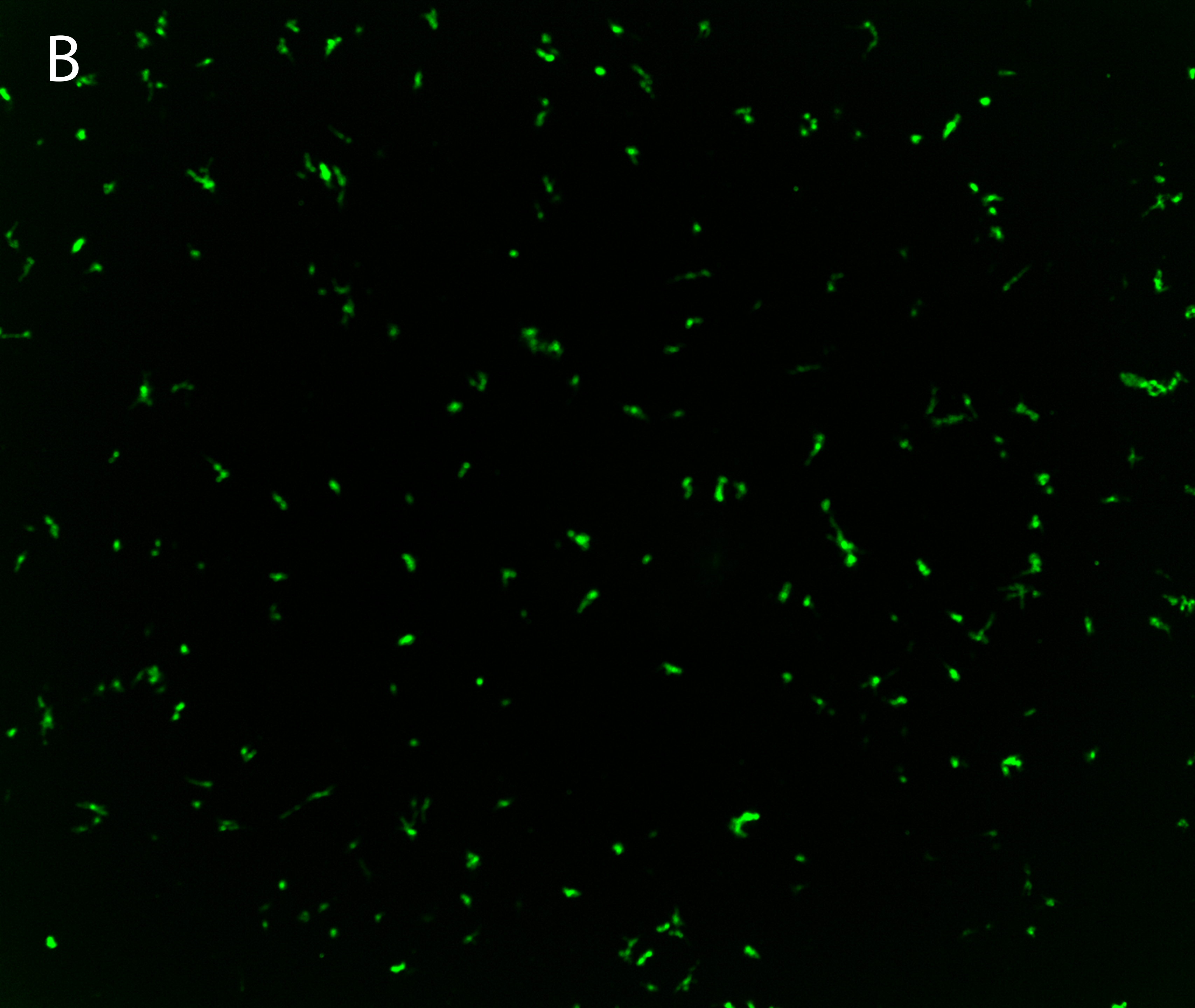

Supplement: Supplemental material [file CVI.00225-17_zcd012175545s2.pdf]
