# Supplementary material for: Development of a High-Throughput Respiratory Syncytial Virus Fluorescent Focus-Based Microneutralization Assay
Source: Clin Vaccine Immunol. 2017 Dec 5;24(12):e00225-17. doi: 10.1128/CVI.00225-17 (PMC5717189; doi:10.1128/CVI.00225-17)
Supplement: Supplemental material [file CVI.00225-17_zcd012175545s3.pdf]

Development of a High-Throughput Respiratory Syncytial Virus Fluorescent Foci-Based Microneutralization Assay

Shambaugh C, Sarieh Azshirvani S, Yu L, Pache J, Lambery SL, Zuo F, Esser MT

Supplementary figure legends

Figure S1. RSVA-GFP virus growth curve in Vero cells as measured by fluorescent foci in 96-well plates using the IsoCyte™ laser imager. Images of a virus only well were collected at 10 time points over a 48 hour period. The black filled circles represent the linear range of the curve spanning from 15 – 25 hours post infection. Foci appear to decrease in numbers after 28 hpi, however, plate well images revealed that the foci had increased in size and fused with neighboring foci which demonstrated cell spread from initial infection and was not due to cell loss.

Figure S2. Screen shots from the ImageXpress Micro MetaXpress software showing fluorescent foci per region of interest (ROI). (A) Image of GFP-RSV infected Vero cells at 22 hpi with 259 foci/ROI; (B) Image of the same well at 22 hpi after acetone fixation and immunostaining with 250 foci/ROI.
